# Using the Indocyanine Green (ICG) Lymphography to Screen Breast Cancer Patients at High Risk for Lymphedema

**DOI:** 10.3390/diagnostics12040983

**Published:** 2022-04-14

**Authors:** Miao Liu, Siyao Liu, Quanping Zhao, Ying Cui, Jin Chen, Shu Wang

**Affiliations:** Breast Center, Peking University People’s Hospital, Beijing 100044, China; liumiao@pkuph.edu.cn (M.L.); dr.liusiyao@pku.edu.cn (S.L.); zqp030424@sina.com (Q.Z.); cuiying1848@163.com (Y.C.); chenjinwy@163.com (J.C.)

**Keywords:** breast cancer, lymphedema, indocyanine green (ICG) lymphography

## Abstract

Background: Indocyanine green (ICG) lymphography is a newer technique for diagnosing lymphedema. Our study aimed to find whether the abnormality of ICG lymphography can predict the occurrence of early lymphedema and then select candidates at high risk of developing lymphedema. Methods: Postoperative breast cancer patients who visited the lymphedema clinic of Peking University People’s Hospital from December 2016 to September 2019 were consecutively enrolled and received ICG lymphography and circumference measurement. Data were collected on the patients’ characteristics and correlation between ICG lymphography and the occurrence of lymphedema. Results: The analysis included 179 patients. There were 91 patients in the lymphedema group and 88 patients in the non-lymphedema group. By multivariate analysis, age, axillary surgery, radiotherapy, and time since breast cancer surgery were regarded as risk factors for lymphedema (*p* < 0.05). According to the results of ICG lymphography, patients in the non-lymphedema group (*n* = 88) were divided into ICG-positive (*n* = 47) and ICG-negative (*n* = 41) groups. The incidence of lymphedema in the ICG-positive group was significantly higher than that in the ICG-negative group (19.1% vs. 2.4%, *p* = 0.027). Conclusion: Lymphatic disorder can be detected before circumference change using ICG lymphography. Abnormal ICG lymphography is an independent risk factor for lymphedema. Patients with abnormal dermal backflow patterns are considered to be a high-risk group for lymphedema and should undergo early interventions to prevent lymphedema.

## 1. Introduction

Breast cancer-related lymphedema is a common complication following breast cancer treatment, which severely affects patients’ quality of life. The treatment of lymphedema has always been difficult. This may be because, when most patients are diagnosed with lymphedema, the lymphedema is already serious, and their upper limb lymphatic system has undergone a chain of complex and progressive pathological remodeling [1]. Identifying subclinical lymphedema is helpful for early treatment and may reduce the chance that the disease will progress to a chronic late stage. Early identification and treatment of lymphedema can provide greater treatment success rates and potential cost savings [2]. Therefore, sensitive lymphedema screening tools are necessary and can detect early lymphedema in time.

The commonly used physical methods for diagnosing lymphedema (such as arm circumference measurement (CM) and volume measurement) are not sensitive to changes in the early stages of lymphedema and are easily affected by tissues that may change independently from lymphedema (such as muscle and fat) [3]. Bioimpedance spectroscopy (BIS) is considered to have a good ability to detect subclinical lymphedema [4,5]. However, it has many shortcomings, such as unobjective values and large fluctuations (pressure, temperature, and daily activities will affect the results of BIS) [6,7]. In addition, its sensitivity remains disputed owing to the wide range of sensitivities observed (30–100%), and a study has reported a high rate of false-negative results [6]. All of these problems question the ability of BIS to detect the disease early.

In the past decade, imaging of the peripheral lymphatic vasculature has been developed, which can offer a potential new way to detect lymphatic disruption before signs of lymphedema become visible. The traditional standard imaging method used to image the lymphatics is lymphoscintigraphy. Although it has been widely used, lymphoscintigraphy has many disadvantages that limit clinical and research uses, including the use of radiotracers and relatively poor spatial resolution [8]. More recently, indocyanine green (ICG) lymphography is a newer technique that has a higher sensitivity and specificity than lymphoscintigraphy [7,9]. This technique allows for quick pathological visualization of superficial lymph flow in real time, without radiation exposure. It can accurately and reliably diagnose, track, and stage the severity of lymphedema, ranging from subclinical or early lymphedema to more advanced cases [7]. 

At present, most research focused on the application of ICG lymphography to patients with definite lymphedema and use it for the visualization and analysis of lymphatic vessels. However, there are few studies on ICG lymphography as a diagnostic method for lymphedema, and the sample size of these studies is small, while the follow-up time is short. In addition, there is still a lack of more clinical data to analyze the relationship between abnormal ICG lymphography and the occurrence of early lymphedema. Therefore, the evidence for clinical application of ICG lymphography is insufficient. In this study, we applied ICG lymphography in patients who have not yet developed clinical lymphedema to explore the influence of abnormalities of ICG lymphography on the occurrence of lymphedema, which aim to provide good data support for the future clinical application of ICG lymphography.

## 2. Methods

### 2.1. Patients

This study was a retrospective clinical trial conducted at the Peking University People’s Hospital (PKUPH). Approval from the ethics committee of PKUPH was obtained. Postoperative breast cancer patients who visited the lymphedema clinic of Peking University People’s Hospital from December 2016 to September 2019 were consecutively enrolled and received ICG lymphography and circumference measurements. Patients with bilateral breast cancer and who were lost to follow-up were excluded. 

All patients were divided into lymphedema and non-lymphedema groups based on the results of CM. Patients in lymphedema group received standard treatment. Patients in the non-lymphedema group were followed up using CM.

### 2.2. ICG Lymphography

All patients received an ICG lymphography with the same protocol at the first visit. ICG (0.2 mL, Dandong Pharmaceutical, Jilin, China) was injected subcutaneously into the distal affected upper extremity at the second web space of the dorsal aspect of the hand. Image recording began immediately after injection. An immediate scan and every 30 min scan was acquired using the Photodynamic Eye infrared camera system (MingDe Biomedical Technology, China). ICG lymphographic images were classified into a linear pattern, three dermal backflow patterns (splash, stardust, and diffuse). The presence of dermal backflow pattern is considered an abnormality of ICG lymphography. ICG lymphography was classified as stages 0–5 by M. D. Anderson classification (MDACC) staging [10].

### 2.3. Lymphedema Assessment and Follow-Up Data Collection

Patients with positive arm CM received interventional treatment. Patients with negative arm CM did not receive interventional treatment regardless of the results of ICG lymphography and completed a specific follow-up visit to check for the occurrence of any breast cancer-related lymphedema (BCRL). Follow-up visits were scheduled at 3, 6, 9, and 12 months after the first visit for the first year and then every half year thereafter. The median follow-up period was 25.20 (range, 5.03–45.24) months. In our study, the arm circumference was measured at ten sites on both limbs using a tapeline measurement. The locations measured were mid-metacarpal; the wrist; at 5 and 10 cm above the wrist; elbow fold; at 10 and 5 cm below the elbow fold; and at 15, 10, and 5 cm above the elbow fold [11]. All measurements were performed by the same investigator. Lymphedema was defined as a 2 cm or greater increase in ipsilateral arm measurements compared with contralateral arm measurements at any of the 10 measured locations on the limb [12]. 

### 2.4. Statistical Analyses

All analysis was carried out using SPSS statistical software, version 24.0. (IBM Corp., Armonk, NY, USA). Continuous data are presented as the mean values  ±  standard deviation. Associations between patients’ characteristics and lymphedema status were examined using the univariable logistic regression and multivariable logistic regression. Univariable COX regression was used to analyze the incidence of lymphedema, as well as the factors affecting the occurrence of lymphedema in the ICG-positive group and ICG-negative group. Association between the patients’ arm circumference difference and ICG staging was examined using the Kruskal–Wallis H test. A “*p*-value” less than 0.05 was considered statistically significant.

## 3. Results

### 3.1. Patients

**Figure 1:**
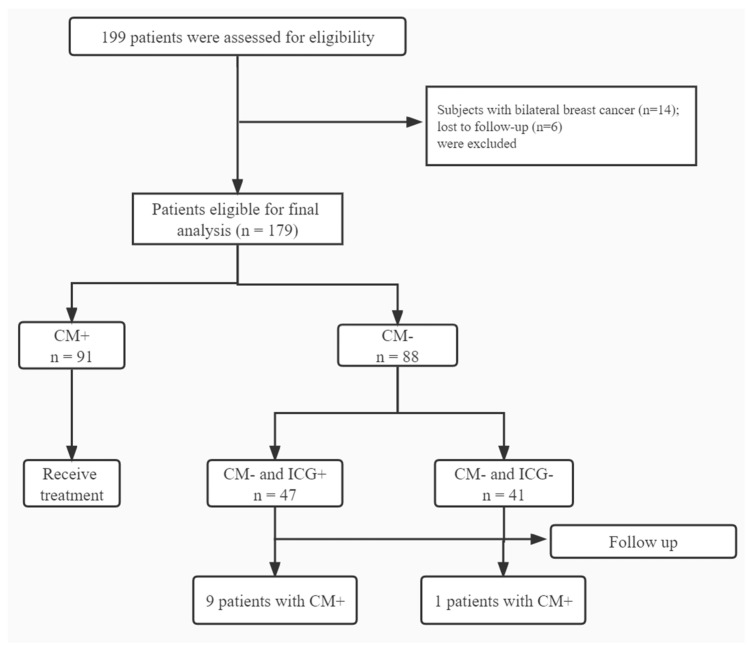
Flowchart of the process of patients’ enrollment (ICG, indocyanine green; CM: circumference measurement).

There are 199 postoperative breast cancer patients who visited the lymphedema clinic of Peking University People’s Hospital from December 2016 to September 2019 were consecutively enrolled and also completed ICG lymphography and CM. After elimination, a total of 179 patients were included in our study from December 2016 to September 2019. The bilateral arm circumference difference of 91 patients were greater than 2 cm, which met the definition of lymphedema and belonged to the lymphedema group. The remaining 88 patients’ arm circumference difference were less than 2 cm and belonged to the non-lymphedema group. According to the results of ICG lymphography, patients in the non-lymphedema group (*n* = 88) were divided into ICG-positive (*n* = 47) and ICG-negative (*n* = 41) groups (Figure 1) and received follow-up by the circumference method. The 88 patients in the non-lymphedema group did not receive interventional treatment, but only received routine education. Another 91 patients in the lymphedema group received treatment. 

### 3.2. Correlation between Clinicopathological Features and Lymphedema

There were 91 patients in the lymphedema group and 88 patients in the non-lymphedema group. Participant demographic characteristics are presented in Table 1. Using univariate analyses, significant differences were found in the age, breast surgery, axillary surgery, chemotherapy, radiotherapy, and time since breast cancer surgery between the two groups. However, there were no differences in the body mass index (BMI) and affected limb between the two groups. By multivariate analysis, age, axillary surgery, radiotherapy, time since breast cancer surgery remained significantly predictive of lymphedema (*p* < 0.05) (Table 2).

### 3.3. Analysis of ICG Lymphography

**Figure 2:**
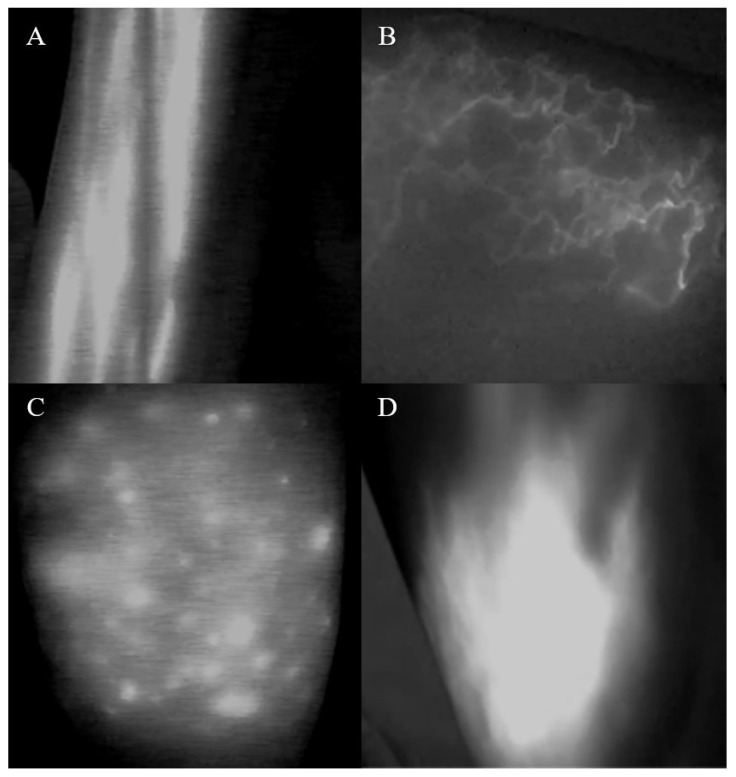
Normal pattern and dermal backflow patterns of ICG lymphography ((**A**): linear pattern; (**B**): splash pattern; (**C**): stardust pattern; (**D**) diffuse pattern) (ICG, indocyanine green).

ICG lymphographic images were classified into a linear pattern, three dermal backflow patterns (splash, stardust, and diffuse) (Figure 2). One or more patterns may appear on the same patient. All patients in the lymphedema group had abnormal ICG lymphography, while 47 patients in the non-lymphedema group had abnormal ICG lymphography. 

**Figure 3:**
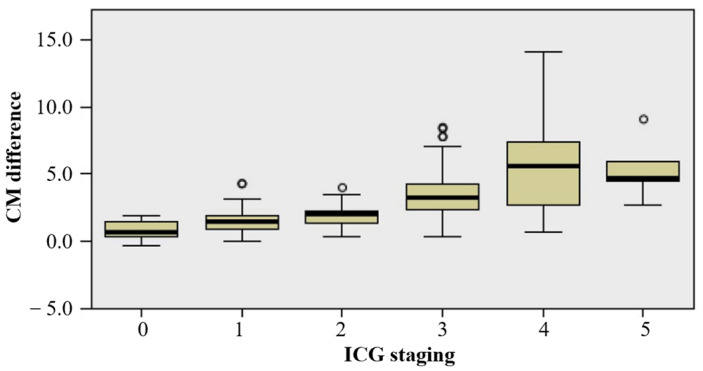
The distribution of CM difference between different ICG stages (ICG, indocyanine green; CM: circumference measurement).

In addition, ICG lymphography could also be classified as stages 0–5 by MDACC staging [10]. There are 41, 31, 25, 52, 24 and 6 patients in stages 0, 1, 2, 3, 4, and 5, respectively. We compared the differences in the distribution of arm circumference difference between different ICG stages and found that the distribution of arm circumference difference in each group was not all the same, and the difference was statistically significant (H = 98.771, *p* < 0.001) (Figure 3). The median arm circumference differences for ICG staging 0–5 are 0.7 cm, 1.5 cm, 2.0 cm, 3.3 cm, 5.6 cm, 4.7 cm, respectively. The median of the total arm circumference difference is 2 cm.

All patients who have undergone ICG lymphography had no complications, such as infection, subcutaneous necrosis, allergies, etc. The subcutaneous staining at the injection site disappeared in about 1 week.

### 3.4. Using ICG Lymphography to Predict the Occurrence of Lymphedema

According to the results of ICG lymphography, patients in the non-lymphedema group (*n* = 88) were divided into ICG-positive (*n* = 47) and ICG-negative (*n* = 41) groups. After a median 25.20 months’ follow-up, there were nine and one patients developed lymphedema in these two groups, respectively. The incidence of lymphedema in the ICG-positive group was significantly higher than that in the ICG-negative group (19.1% vs. 2.4%, *p* = 0.027). Therefore, we thought that ICG lymphography is an independent risk factor for the occurrence of lymphedema (Table 3). Other clinicopathological factors such as age, BMI, affected limb, breast surgery, axillary surgery, chemotherapy, and radiotherapy cannot predict the occurrence of lymphedema.

## 4. Discussion

In 2007, ICG lymphography was first introduced to assess subcutaneous lymphatic function, which is a less invasive alternative to lymphoscintigraphy [13]. In 2011, Yamamoto, T. et al. [14] used ICG lymphography for the first time to observe abnormal images of the lymphatic drainage of the upper limbs and tried to analyze the relationship between this abnormality and the severity of lymphedema. They established a new severity staging system for upper extremity lymphedema based on changes in 20 patients’ ICG lymphographic findings—that is, the arm dermal backflow pattern. Then, they formulated the concrete arm dermal backflow stages (stage 0-stage V), based on the severity of the dermal backflow and the location where it appears in the arm. However, we found that their staging system is complicated and not completely applicable in actual clinical work, especially for patients who have only local dermal backflow pattern. In 2013, David W. et al. [10] developed a simple, user-friendly, clinically relevant staging system of lymphedema using ICG lymphography that was called MDACC staging. Therefore, in our study, ICG lymphography was classified as stages 0–5 by MDACC staging [10]. Additionally, we found abnormal ICG lymphography, such as splash, stardust, and diffuse pattern in our study. 

However, what is the relationship between abnormal ICG lymphography and lymphedema? At present, there are not many studies on this aspect. In 2016, Akita, Shinsuke et al. [15] reported the results of a prospective study involving 196 breast cancer patients, showing that the abnormalities of ICG lymphography would appear ahead of the occurrence of clinical lymphedema. However, in their study, when the stardust, diffuse, and no flow pattern appeared, they would start interventional treatment, failing to prove the relationship between abnormal dermal backflow and clinical lymphedema due to the early intervention. Therefore, we want to use our study to make some better supplements for the diagnosis of lymphedema by ICG lymphography. In our study, we observed the natural progression of patients with abnormal ICG lymphography and analyzed the relationship between the abnormal ICG lymphography images and the occurrence and change of lymphedema. In our study, all patients in the lymphedema group had abnormal ICG lymphography, which proved the ability of ICG lymphography in diagnosing lymphedema. 

However, whether the abnormalities of ICG lymphography can predict the occurrence of lymphedema is unclear. As far as we know, there is no particularly good method to predict the occurrence of lymphedema nowadays. BIS may have ability to detect early-stage lymphedema, but it has many shortcomings, such as unobjective values and large fluctuations, as well as high false negative rates. MR lymphangiography [16,17] may also be valuable in detecting early lymphatic abnormalities, but more suitable contrast agents and sequences is still explored. At present, doctors mainly judge the high-risk population of lymphedema based on clinicopathological factors, but these factors are difficult to objectively predict the occurrence of lymphedema. Since ICG lymphography has the ability to directly visualize lymphatic vessels, some scholars try to use this method to predict the occurrence of lymphedema.

In our study, we found that patients in the non-lymphedema group (*n* = 88) could be divided into ICG-positive (*n* = 47) and ICG-negative (*n* = 41) group. After a median 25.20 months follow-up, there were nine and one patients developed lymphedema in these two groups, respectively. This proved the ability of ICG lymphography in predicting lymphedema. Other clinicopathological factors failed to show the ability to predict the occurrence of lymphedema. Therefore, we thought that ICG lymphography is an independent risk factor for the occurrence of lymphedema. Although ICG lymphography is invasive, we did not find any side effects due to the small amount of injection. To our knowledge, this is the first study to explore the influence of abnormalities of ICG lymphography on the occurrence of lymphedema. More large sample studies are needed to confirm our findings. 

In addition, our data also found that there seems to be a correlation between MDACC staging and the severity of lymphedema. In fact, regardless of the Yamamoto’s staging or MDACC staging, the appearance of abnormal dermal backflow pattern is regarded as the abnormal performance of ICG lymphography, and they just explored different staging methods based on the experience of their own center. In the future, a more optimal staging remains to be explored. The treatment of lymphedema is very difficult. The reason is that most of the patients who come to the clinic have already developed obvious lymphedema. Although we believe that early diagnosis and treatment is definitely beneficial to lymphedema, the most critical difficulty is to screen patients in need of treatment. 

As we all know, traditional risk factors related to lymphedema have been reported in the published literature, including mastectomy, axillary lymph node dissection (ALND), regional lymph node irradiation, higher BMI, and older age at diagnosis [18,19]. In our study, we have similar conclusion. If treatment is given to all patients with high risk factors for lymphedema, then the treatment is undoubtedly excessive and does not conform to health economics, and patient compliance is also poor. Therefore, we especially need a tool that can screen high-risk patients of lymphedema and then give targeted and precise treatment to the selected people. Our research has found the value of ICG lymphography in predicting lymphedema, so the early treatment based on the guidance of ICG lymphography is the direction of our follow-up work.

There are some limitations associated with our study, including retrospective design, the small sample size, single-center design, and short follow-up times. In addition, our study included postoperative breast cancer patients who visited the lymphedema clinic, which may have resulted in selection bias regarding disease severity. 

The meaning of our research is to observe the natural process of abnormal ICG lymphography and analyze the relationship between it and the occurrence of lymphedema, so as to provide evidence to support the diagnosis of lymphedema by ICG lymphography. It is an important supplement to the Japanese research.

## 5. Conclusions

ICG lymphography is a safe and convenient method for evaluation of breast cancer-related lymphedema. Lymphatic function disorder can be detected before circumference change using ICG lymphography. Abnormal ICG lymphography is an independent risk factor for lymphedema. Patients with abnormal dermal backflow pattern are considered to be a high-risk group for lymphedema and should undergo early intervention to prevent lymphedema. Prospective studies are needed to verify the effect of early diagnosis and treatment of lymphedema under the guidance of ICG lymphography.

We believe that the results of our research in this article can provide data support for the future clinical application of ICG lymphography.

## Figures and Tables

**Table 1:** Univariable logistic regression of participant characteristics.

Variable	Lymphedema Group (*n* = 91)	Non-Lymphedema Group (*n* = 88)	*p*-Value
Age (years)	60.43 ± 11.56	54.20 ± 11.47	0.001
BMI (kg/m^2^)	25.02 ± 3.52	25.12 ± 3.29	0.841
Affected limb			0.600
Right	44	46	
Left	47	42	
Breast Surgery			0.001
BCS	11	30	
Mastectomy	80	58	
Axillary surgery			0.000
SLNB	1	31	
ALND	90	57	
Chemotherapy			0.030
Yes	86	74	
No	5	14	
Radiotherapy			0.011
Yes	71	53	
No	20	35	
Time since BC surgery (months)	67.45 ± 85.72	22.48 ± 18.39	0.000

BMI, body mass index; SLNB, sentinel lymph node biopsy; ALND, axillary lymph node dissection; BCS, breast-conserving surgery; BC, breast cancer.

**Table 2:** Multivariable logistic regression of clinicopathologic data.

Characteristic	β	S.E	*p*	OR	95% CI
Age	0.042	0.019	0.026	1.043	1.005–1.082
Breast Surgery	−0.674	0.534	0.207	0.510	0.179–1.452
Axillary surgery	3.564	1.131	0.002	35.308	3.849–323.874
Radiotherapy	1.274	0.457	0.005	3.574	1.460–8.746
Chemotherapy	−0.512	0.797	0.520	0.599	0.126–2.856
Time since BC surgery	0.024	0.008	0.004	1.024	1.008–1.041

BC, breast cancer; CI, confidence interval; OR, odds ratio.

**Table 3:** Univariable COX regression of the risk factors affecting lymphedema.

Variable	No.	Events	Mean Follow-Up without LE (Months)	*p*-Value	HR	95% CI
Group						
ICG+	47	9	22.06	0.027	10.437	1.313–82.994
ICG−	41	1	29.02			
Age (years)						
≥60 years	29	5	25.51	0.311	1.904	0.548–6.611
<60 years	59	5	25.20			
BMI (kg/m^2^)						
≥24	54	9	23.04	0.068	6.904	0.869–54.832
<24	34	1	28.90			
Affected limb						
Right	46	8	24.93	0.099	3.680	0.781–17.334
Left	42	2	25.71			
Breast Surgery						
Mastectomy	58	9	23.80	0.107	5.489	0.694–43.415
BCS	30	1	28.20			
Axillary surgery						
ALND	57	10	24.87	0.159	44.630	0.225–8859.097
SLNB	31	0	26.10			
Chemotherapy						
Yes	74	10	24.23	0.325	29.056	0.035–23894.466
No	14	0	30.98			
Radiotherapy						
Yes	53	9	25.51	0.086	6.106	0.773–48.200
No	35	1	24.99			

ICG, indocyanine green; BC, breast cancer; CI, confidence interval; HR, hazard ratio; LE, lymphedema; BMI, body mass index; SLNB, sentinel lymph node biopsy; ALND, axillary lymph node dissection; BCS, breast-conserving surgery.

## Data Availability

All data generated or analyzed during this study are included in this article.

## Abbreviations

ICG: indocyanine green; BIS: bioimpedance spectroscopy; PKUPH: Peking University People’s Hospital; CM: circumference measurement; MDACC: M. D. Anderson classification; BCRL: breast cancer-related lymphedema; BMI: body mass index; SLNB: sentinel lymph node biopsy; ALND: axillary lymph node dissection; BCS: breast-conserving surgery; BC: breast cancer; CI: confidence interval; OR: odd ratio; LE, lymphedema.
